# Memory in performance: kinesthetic and procedural dimensions of skill acquisition in dance improvisation

**DOI:** 10.3389/fpsyg.2026.1751590

**Published:** 2026-03-13

**Authors:** Diego Marin-Bucio, Anne Danielsen, Noumouke Doumbia, Rainer Polak

**Affiliations:** 1 RITMO Center for Interdisciplinary Studies in Rhythm, Time, and Motion, University of Oslo, Oslo, Norway; 2 Department of Musicology, University of Oslo, Oslo, Norway; 3 Département des Sciences de l'Éducation, Université Yambo Ouologuem, Bamako, Mali

**Keywords:** autoethnography, autophenomenology, dance improvisation, dance learning, embodiment, kinesthetic memory, procedural memory

## Abstract

Improvisation is central to creative behavior across artistic and everyday domains, yet it is often portrayed as either pure freedom or rule-bound execution. While research in music and dance has shown that improvisation draws on structured kinesthetic vocabularies, less is known about how cultural rhythm and embodied memory interact in real time within and across genres. This study addresses that gap through ethnographic fieldwork in West Africa, where the first author, trained in contemporary dance, engaged in learning and performing Malian djembe dance. Drawing on autoethnography with a phenomenological orientation, alongside participant observation and conversations with Malian drummers and dancers, the analysis examines how kinesthetic and procedural memories inform real-time performance. Findings suggest that improvisation operates through culturally specific ways of sensing and attending to movement: dancers navigate genre-specific repertoire, rhythmic cues, and bodily affordances to evoke and transform embodied material. However, rather than merely reproducing fixed repertorial *units*, dancers also reconfigure embodied resources such as movement *qualities* in responsive and inventive ways. Our research supports the view of improvisation as structured play rather than unbound invention and advances the discourse by emphasizing the reconstructive play of embodied recall—how cultural and personal memories are recomposed in performance. Overall, the study contributes to understanding improvisation as a cognitive and cultural process: not the free invention of form but the creative reorganization of embodied memories within shared perceptual and rhythmic systems.

## 1 Introduction

What allows my body^1^ to create movements that seemingly arise on their own, rather than being dictated by conscious instruction? As a practitioner of contemporary dance, I regularly confront this fundamental question after spending long hours improvising in the studio, recording my sessions to later observe how my movement unfolds. During these explorations, not everything that emerges is interesting, yet I do encounter moments of striking fluency when thought recedes and I enter a state that I experience as meditation in movement.

I record these sessions as part of my creative process because I trust the intelligence of my intuition as it manifests through improvisation. In those moments of dancing, my attention can be completely absorbed in the movement itself. When I look back upon such moments with the help of the recordings, I try to recall what I was thinking, but no clear images or words return—only the sensation of flow (see Gang and Zhang, 2024).

In the context of contemporary dance,^2^ certain improvisational practices cultivate access to this suspended mode of thinking, where the dancer is not moving but being moved (see Sakuta, 2020, pp. 67–72). It intrigues me how my body navigates this state and executes split-second transitions with such fluency that action appears to precede intention. The same occurs when I improvise before an audience—I step into the unknown, trusting that whatever emerges will remain coherent with the unfolding present.^3^

These experiences resonate with a broader human condition: improvisation understood as spontaneous creation is not confined to the arts but pervades everyday life. We improvise continually—in conversation, in navigating unpredictable situations, in adapting to others' gestures and emotions. As Ingold and Hallam (2021) suggest, improvisation is a fundamental mode of human engagement with the world, one through which meaning, coherence, and connection are continuously generated. Studying dance improvisation thus provides a specific lens for examining the microstructures of this embodied intelligence—how perception, memory, and action intertwine in real time to produce sense without a preexisting plan.

The present paper is based on ethnographic fieldwork with dancers and drummers in West Africa, focusing on a dancer trained in contemporary dance—first author Marin-Bucio—who learned to perform djembe dance from Mali. Skill acquisition and improvisation across the performance and learning processes are explored both through the learner's autoethnographic, phenomenological perspective and the insights of the Malian fieldwork partners who acted as tutors.

Djembe dance performance offers a suitable context for studying dance improvisation. Its social prominence and longstanding professionalization make it relatively accessible to ethnographic and artistic research, while the extensive scholarship on its musical—and, to a lesser degree, its dance—components provides a solid foundation for further inquiry. Existing research on improvisation in dance has largely focused on Euro-American genres, a bias our African case study helps to expose and redress. Whereas contemporary dance often emphasizes its autonomy from music, djembe performance is founded on the deep integration and co-evolution of its music and dance, so that it represents a coherent music-dance genre (Haugen, 2016). Such forms whose expressive modes are strongly interdependent are in fact common across cultures and historical periods; thus, this study provides a valuable framework for examining improvisation around the world and throughout time.

The paper's objective is twofold. First, we aim to identify key elements mobilized in improvisatory play in different dance traditions. We ask how improvisation in dance unfolds—what it plays *with*—and to what extent this is contingent on the specific genre in question. This step is essential for recognizing and describing improvisatory processes as such. The second objective is to understand the conditions and mechanisms that enable dancers to use kinesthetic vocabulary creatively. What are the prerequisites and procedures at work when dancers mobilize the materials with which they improvise?

### 1.1 Improvisation in music and dance studies

Pearson (2021) examines the concept of “improvisation” in music studies, highlighting its problematic status shaped by colonial histories that have prioritized composition and marginalized improvisatory practices. Rejecting the binary of “composed” vs. “improvised,” she argues that musical practices combine fixed elements and elements open to play, this play in turn presupposing materials recognizable to both coperformers and audiences. Much research emphasizes repertorial units, such as motifs^4^ and phrases^5^ (e.g., jazz “standards”; Faulkner and Becker, 2009; see also Cochrane, 2000; Cook, 2007), yet non-representational features—proprioceptive sensations, shifts in weight and energy, and subtle movement qualities—also serve as improvisatory material in contemporary dance (Sarco-Thomas, 2019). Importantly, Pearson points out, “the features that are fixed and at play can vary from style to style, format to format, and any given musical element (phrase or process) may show fixity at some levels and play at others” (Pearson, 2021, p. 447).

In Euro-American dance, improvisation was long overshadowed by choreographic permanence but resurfaced during key aesthetic shifts, from early modern dance's embrace of spontaneity (Daly, 1994) to the postmodern experimentation of the 1960s (Novack, 1990; Paxton, 1975). Over time, it gained recognition as both artistic method and social intervention practice within therapeutic, educational, and academic contexts (Novack, 1990; Pallant, 2006). Research in these domains emphasizes the epistemological challenge of writing about dance improvisation. Language generally struggles to convey its non-linear, multidimensional nature (De Spain, 2014) and experiental immediacy (Blom and Chaplin, 1988), as well as related sensations such as fleeting shifts in weight, speed, flow, and other movement qualities.

In African dance studies, improvisation has been remarked upon as a constitutive element of both social and staged dance. Gottschild (1996) and Daniel (2005) emphasize how improvisation is embedded in African diasporic dance traditions, where call-and-response structures, rhythmic play, and individual variation are central to the aesthetic. More recent research examines improvisation in West African contexts. Seye (2019) studies social *sabar* dance events in Senegal as significant spaces where participants negotiate cultural norms, values, and identities through performance. She demonstrates that these events reinforce communal ideals by balancing individual expression with adherence to tradition, enabling improvisatory interaction within a framework of shared social meaning.

### 1.2 Improvisation in djembe music and dance

Rooted in the cultural traditions of Mande-speaking societies of northeastern Guinea and southern Mali, djembe music has served as the rhythmic foundation for local dance celebrations for centuries. Today, the genre's evolution reflects a feedback loop across three primary performance contexts that continue to coexist and coevolve (Polak, 2000). In local celebrations, djembe performance remains embedded in family and community festivities (Polak, 2021, in press). Pan-African and then national staged dance-theater cultures (*ballets africains*) emerged starting in the 1950s with the independence movement; in the 1960s, newly independent Guinea and Mali staged djembe music and dance ensembles as a national symbol through choreographic arrangements, formalized rehearsals, and virtuosic performance standards (Djebbari, 2012, 2019). From the 1980s onward, djembe music migrated globally, developing its own transnational performance scene (Charry, 2000; Polak, 2000; Zanetti, 1996).

Each context has shaped the genre's music and dance characteristics, social functions, and cultural meanings. The national ballet context is designed to impress a separate audience (Djebbari, 2012, 2019), while local celebrations frame music as a call to, and accompaniment of, dance, allowing community members to assume the role of the dancer and partake in the collective festivity (Polak, 2021, in press). Local and national contexts deeply integrate music and dance, supporting conceptualizations of djembe performance as a music-dance genre (Haugen, 2016), whereas the global diffusion has increasingly favored an emancipation of the percussive art from the dance.

In local celebration contexts, djembe dance in circle formations is coregulated: sound and kinetic rhythms are spontaneously negotiated through recognizable cues. Improvisation is dialogic and dancer led; a dancer entering the circle adjusts motifs to the ongoing drumming, yet their performance can compel the lead drummer to adapt or even change the repertoire on the fly (Djebbari, 2019; Polak, 2007, 2021; in press). This aligns with Nzewi's (1991) notion of “performance composition,” wherein rhythmic creativity emerges through situated recomposition, and with Moreno and Moreno's (1972) concept of spontaneity as readiness to act appropriately and inventively.

By contrast, the adaptation of traditional dances for the stage in the dance-theater context reshaped the forms and functions of improvisation. The spontaneous negotiation of solos, central in local celebrations (Polak, 2007, 2021, in press), was minimized in the staged ballets, where choreography followed prearranged sequences. Structured percussive cues synchronized *corps de ballet* performances, prioritizing visual order over individual improvisation, and this principle remains vital in many Malian genres (Djebbari, 2012; Polak, 2004; Zanetti, 1996).

### 1.3 Research objectives and key concepts

The research reviewed above demonstrates that improvisation functions both structurally and socially, negotiating agency, power, and creativity among dancers, musicians, and, in staged contexts, artistic directors and audiences. Historical developments show that improvisation cannot be treated as a universal or purely aesthetic phenomenon; its structures, functions, and meanings shift across cultural and institutional settings, challenging Euro-American notions of improvisation as a context-free procedure. This underscores the first objective of the present paper: to identify key elements in spontaneous performance as a first approximation of improvisatory structures. The second objective—examining the prerequisites and procedures by which dancers mobilize materials moment-to-moment—has, to our knowledge, remained unaddressed in studies of West African dance to date. In filling this gap, the present research offers its principal novelty.

Our research defines improvisation as the playful, spontaneous mobilization of genre-typical parameters or fixed elements during performance, unfolding in real time. Critically, these elements must be at the performer's disposal in the moment of performance. We posit that, for this to be possible, three components are required. First, the elements must have evolved historically and have been established through social practice over periods of time—that is, they must preexist the performance itself.^6^ Second, performers must have acquired the perceptual and motor skills to recognize and enact these elements through processes of social learning during enculturation, typically gained by participating in these very practices. Third, for these cultural products or processes and individual skills to be available to performers, they must be embodied and maintained over time; in this context, memory plays a critical role.

#### 1.3.1 Kinesthetic memory and kinesthetic consciousness

**Figure 1:**
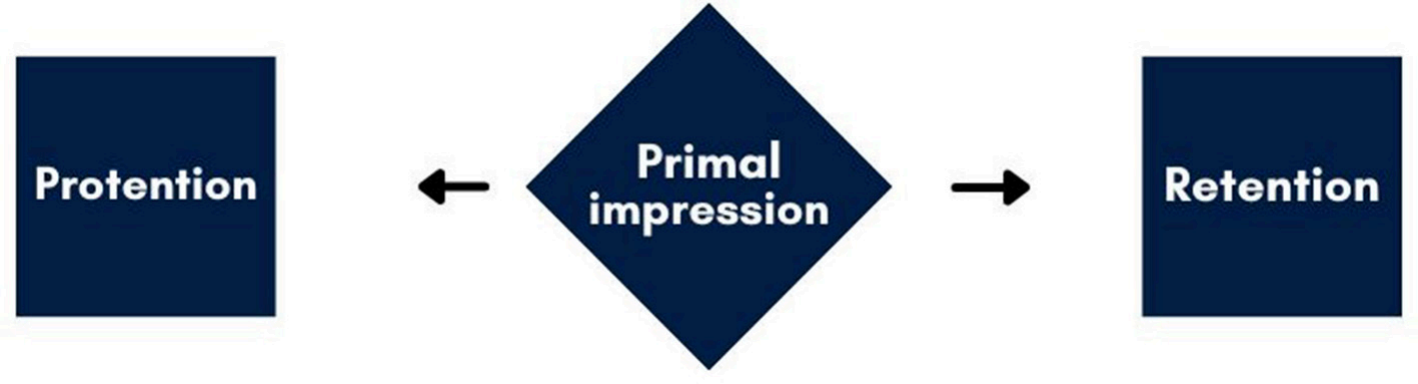
Schematic representation of unified flow of consciousness according to (Husserl 1991). The flow of protention ← primordial impression → retention directly governs the sense of continuous movement. Protention intends or anticipates the immediate future. It is an automatic, passive component of the living present that sets up an expectation of what will come next. Retention is the aspect of consciousness that makes us aware of the immediate past. It is an automatic, passive way of holding onto what has just occurred.

Kinesthetic memory forms a key nexus between culturally transmitted, socially learned genre-specific elements and their mobilization by individual dancers in improvisatory performance. It builds on kinesthetic consciousness, which we understand in line with philosopher Maxine Sheets-Johnstone as an indivisible lived kinetic process (Sheets-Johnstone, 2012, pp. 130–134). This process is experienced qualitatively and, when found in self-movement, appears as a felt-unfolding kinetic flux (p. 131). In Husserlian terms, kinesthetic consciousness operates with three components: (1) *protention*, the just-future, (2) *primordial impression*, the now, and (3) *retention*, the just-past (Husserl, 1991; see Figure 1).

Protention and retention work alongside the primordial impression—the direct, immediate experience of the present moment. They are correlative aspects of consciousness that allow movement to be experienced as a temporal horizon rather than a series of isolated, disconnected “now-points.” Protentions and retentions are thus not moments in time but temporal dilations that foreshadow and reverberate—“protend” and “retend”—qualitatively. Because they are temporally constituted not in terms of momentary successions as such—in other words, in terms of befores, nows, and afters—but qualitatively in terms of an ongoing global dynamic, kinetic expectations and what we might call kinetic lingering auras are not reducible to past and future nows (Sheets-Johnstone, 2011, p. 131).

Kinesthetic consciousness operates during dance improvisation by continuously constituting the “living present” of movement through the dynamic interplay of retention, primordial impression, and protention. This loop ensures that every “now” of movement contains both its own history and its own anticipated future, resulting in a single, unified, and spontaneous kinetic experience. For example, while dancing, feeling the momentum of the last turn (retention) allows one to execute the balance on one leg *now* (primordial impression) while anticipating the necessity of shifting the weight to move out of the balance (protention).

#### 1.3.2 Thinking through movement and thoughts of movement

Building on this foundation, Sheets-Johnstone (2011) proposes the notion of thinking in movement, which underscores that movement itself is a generative form of thought rather than the mere execution of ideas formed in advance. For her, improvisation makes this quality most evident: dancers are not enacting pre-scripted plans but thinking *through movement* as it unfolds. Spatial pathways, temporal modulations, and dynamic qualities emerge and develop in real time, creating what she terms a *kinetic bodily logos*—a non-discursive form of intelligibility rooted in the lived dynamics of the moving body (pp. 421–423). This kinetic logos is distinct from verbal or symbolic reasoning; it is not translated into propositions but is instead enacted directly in and through corporeal dynamics. Thus, to speak of “thinking in movement” is to affirm that movement is not the product of cognition but a primary mode of cognition itself.^7^

In contrast to this non-discursive kinetic logos, Sheets-Johnstone also acknowledges the presence of what she calls thoughts *of* movement: reflective, concept-mediated moments that arise alongside the ongoing flow of improvisation. These are not the source of movement but brief orienting interventions that enter the dancer's awareness without interrupting the overall kinetic continuity (p. 423). In improvisational contexts, such thoughts might take the form of recognizing a rhythmic cue, deciding to shift level or dynamic quality, or recalling a culturally embedded movement principle. They operate at the edges of the unfolding kinesthetic field, offering intermittent guidance while remaining secondary to the primary, embodied intelligence of thinking in movement.

#### 1.3.3 Connecting the past and the present in improvisation

**Figure 2:**
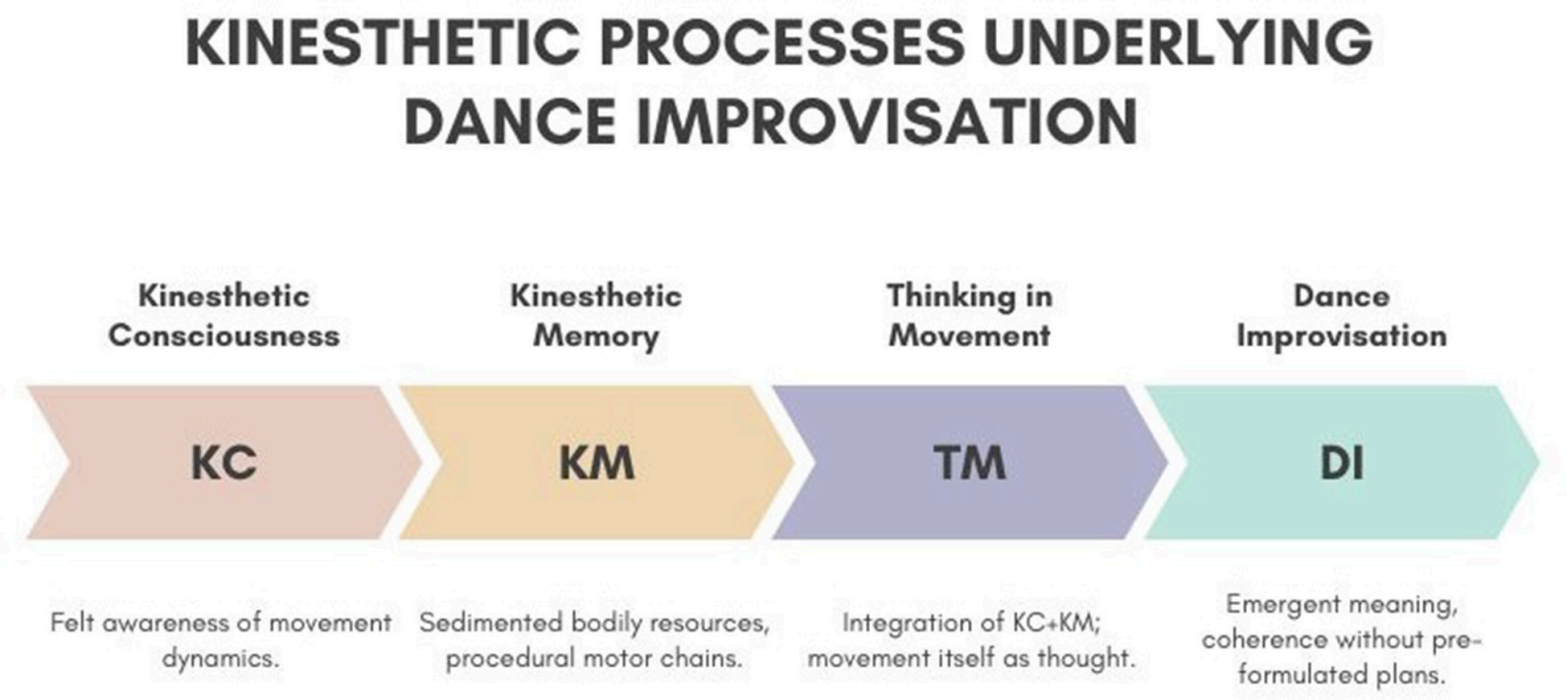
Conceptual model of the kinesthetic processes underlying dance improvisation.

The concept of kinesthetic memory can help to explain how movement improvisation is informed by a dancer's embodied history. Kinesthetic memory is not a matter of recalling fixed representations, such as visual images or verbal instructions, but of reaccessing procedural chains of movement impulses sedimented through practice. This aligns with what cognitive science calls *procedural memory*—the ability to perform learned actions automatically (Sanchez et al., 2010). As Sheets-Johnstone (2015) and others emphasize, kinesthetic memory operates in a prereflective register: one remembers *through doing*, and the memory is triggered by the body's own unfolding action rather than summoned as detached mental content. For instance, beginning the first movement of a familiar phrase can spontaneously “carry” the dancer through its continuation, as one impulse calls forth the next in what Luria (1973) has described as a “kinetic melody.”^8^ In this way, kinesthetic memory is dynamic, flexible, and context dependent: it allows for the recombination, variation, and adaptation of known movement patterns in the flow of improvisation (see Figure 2).

When thinking in movement and kinesthetic memory are considered together, they can illuminate improvisation as an intertwining of the immediate present and the embodied past. Kinesthetic consciousness may ensure the dancer's ongoing awareness of the felt dynamics of movement; kinesthetic memory can supply the sedimented bodily resources accumulated through training and experience; and thinking in movement would designate the *process* by which these are braided into new, emergent movement possibilities. Improvisation, then, can be understood as the dancer's capacity to generate meaning and coherence in action without recourse to preformulated plans, relying instead on the intelligent responsiveness of the moving body itself.

## 2 Materials and methodological approach

### 2.1 Fieldwork setting

The empirical basis for this study is a 6-week fieldwork encounter conducted in October and November 2024. This collaboration involved two researchers from Norway and one from Mali, working together with a group of four Malian djembe performing artists. The fieldwork was carried out in a rented cultural space located near the small town of Kafountine in the Casamance region of southern Senegal.

The fieldwork funding provided by the Research Council of Norway enabled us to offer the participating artists generous financial remuneration, which, from their professional perspective, constituted a familiar and highly valued form of exchange. Inevitably, the intercultural disparity between the Norwegian and Malian subgroups introduced a dimension of power asymmetry characteristic of European–African cultural and research collaborations within the postcolonial world system (Lo and Gilbert, 2002). Nevertheless, this imbalance did not preclude the Malian artists' attainment of social and artistic recognition at both personal and professional levels.

The Malian artists were experienced professionals from Bamako who had specialized in djembe performance since their youth and participated in the three performance contexts described above: local celebration culture, national ballet, and—albeit to a lesser extent—the international scene for djembe drumming in the Global North. The all-male drum ensemble consisted of lead djembe player Issa Traore, lead dundun and accompaniment djembe drummer Massire Camara, accompaniment dundun player Madou Diakite, and accompaniment djembe and dundun drummer Rainer Polak. Since the 1990s, Polak has collaborated with these same Malian artists, among others, in a series of artistic and academic research projects. He also served as the project leader of the present research, working in close partnership with research assistant Noumouké Doumbia, whose contributions as logistical manager and translator—especially during interviews, focus groups, drumming and dance sessions, and informal conversations—were indispensable throughout the fieldwork.

Tenin Malle, a female dance professional from Bamako (b. 1987), played a key role as dance tutor and mentor to the first author, Diego Marin-Bucio, who acted as her temporary apprentice. Marin-Bucio participated in the research as a dance artist, investigator, and learner. His training in contemporary and modern dance informs a practice emphasizing somatic awareness, kinesthetic intelligence, and embodied perception. His embodied expertise in dance, combined with a background in ethnochoreology, shaped his reflexive engagement with djembe dance and his phenomenological, autoethnographic approach to learning and improvisation.

### 2.2 Fieldwork process

Within this setting, we were afforded the opportunity to engage closely with the Malian artists and to participate in daily djembe dance sessions each morning. Although these sessions were mostly pedagogical in nature, the learning process was primarily rooted in embodied observation and repetition, consistent with the informal modes of situated learning commonly found in djembe dance and music traditions, where the transmission of skills and knowledge is fully embedded in the performance practice itself (Polak and Doumbia, 2021).

Each fieldwork day began with a communal breakfast. The housekeepers shared with us fresh, homemade food, and the meal served as our daily briefing—part logistics, part friendly banter that set the tone for the day. We then cleared and arranged the space for dancing and drumming and moved into the approximately 2-h-long morning session. Afterwards, we washed up, rehydrated, and rested before lunch. We ate together, sometimes joined by male members of the local staff. General conversations among all flowed mostly in French, with Noumouké translating as needed; the Malian artists spoke Bambara among themselves, while Rainer, Noumouké, and Diego used English. Afternoon activities alternated between djembe drumming practice, interviews, and focus groups. We closed the day with a shared dinner every evening. Friday afternoons were unscheduled to accommodate the local hosts' and Malian artists' Jumu'ah observance. We reserved one rest day each week without dance, usually Sundays, which we used to take short trips to Kafountine village or a nearby beach.

This process allowed me to learn about ways of coexisting in the local djembe culture. One day, after several sessions spent struggling with a particular step, Tenin pressed a banknote onto my sweating forehead—a spontaneous gesture of congratulation that conveyed encouragement, humor, and acceptance. Such moments revealed how learning in this context extended far beyond pedagogy and movement technique, encompassing aspects of care, recognition, and belonging. In turn, Tenin's curiosity led us to exchange other ways of dancing, giving space to share with her my knowledge in contemporary dance.

### 2.3 Fieldwork roles

Our roles in the field were experiential, not merely observational, with the data deriving from direct engagement with dancing and drumming bodies. In Senegal, our Malian collaborators worked outside their usual contexts in a workshop-like setting, which limited engagement with local performance practice but at the same time offered unique advantages. The pedagogical context inverted the usual researcher–informant hierarchies, positioning the cultural practitioners as teachers and the academic researchers as students and thereby creating agency on both sides. The dance teachers' verbal and gestural corrections revealed practitioners' perspectives, and occasional role reversals—inviting the researcher to lead, for example—enhanced intercultural exchange and aligned the work with artistic research. Crucially, this hybrid ethnographic context emphasized and enabled reflection on the researchers' subjective, lived experiences.

### 2.4 Methodological approach

#### 2.4.1 First-person perspective: autoethnography with a phenomenological orientation

Our fieldwork involved several methods, including interviews, focus groups, and, albeit to only a small extent, participant observation of public performances. However, the core empirical contribution of the present research lies in my (first author Marin-Bucio) repeated participation and immersion in the hybrid pedagogy and performance sessions described above—focusing on teaching and learning but also incorporating artistic performance and sometimes joyful celebration. Due to the ways in which the phenomena in question are enacted, situated, and fundamentally grounded in lived bodily awareness, the study employs an autoethnographic design (Csordas, 1990) with a phenomenological orientation (Sheets-Johnstone, 2015), supplemented by the neurofunctional account of chained motor sequences developed by Luria (1973). Much phenomenological research in artistic performance is productively based on interviews (see, Høffding, 2018). However, in a radically cross-linguistic and cross-cultural research setting such as the present one, such an approach has its limits. Moreover, because improvisational choices emerge within an ongoing, felt present—what Sheets-Johnstone (2011) refers to as the “streaming, qualitative now” (pp. 129–131)—they cannot be meaningfully reconstructed from third-person observation alone. The temporal, rhythmic, and affective textures that give coherence to a dancer's choices are accessible only when also one considers those choices from *within* the act of dancing. External recordings can trace bodily trajectories but cannot render the felt intensities that guide a dancer's micro-decisions (Marin-Bucio and Polak, 2025). I thus decided to rely primarily on autophenomenology,^9^ a first-person approach emphasizing the systematic examination of lived experience as the primary source of insight (Zahavi, 2005). An autophenomenological approach to autoethnography^10^ means accessing prereflective dimensions of experience while remaining accountable to rigorous qualitative research principles. Autoethnography integrates movement journaling, video elicitation, and member checks (periodic consultation with participants to contextualize and reflect on selected interpretations) to document and interpret lived experience. An important part of the interpretation of this material is the “bracketed first-person reflection.” Through phenomenological bracketing—a deliberate shift of attention toward self-somatic experience that allows me to describe how movement feels and unfolds in real time—subjective movement accounts are analyzed as data revealing how perception, intention, and relationality arise within cultural contexts.

While the original notion of phenomenological bracketing ideally sought objectivity (Husserl, 2012 [1913]), this is not fully attainable in the research practice presented here. Specifically, in the present context, phenomenological bracketing does not attempt to erase contextual or cultural influence; rather, it reveals how my contemporary dance background and my emerging djembe vocabulary shaped my attention, action, and decision-making. This influence was examined phenomenologically by observing, *in situ*, how habitual ways of sensing, initiating, and structuring movement informed my responses to rhythmic cues, interactional dynamics, and improvisational choices.

#### 2.4.2 Third-person perspectives

In autophenomenology, embodied experience serves simultaneously as site and instrument of knowledge production (Buckland, 2006). Because a phenomenological autoethnographic approach is grounded in situated self-experience, intersectional and cultural components necessarily shape how movement is perceived, enacted, and interpreted. This situatedness is integral to the method and positions the findings as situated knowledge whose relevance lies in its analytical and theoretical transferability rather than in universal generalization. To contextualize and reinforce such first-person insights, we complemented them with video analysis, member checks, and interviews with Malian dancers and drummers. The video analysis consisted of the observation of how the dance was performed, specifically encompassing the annotation and analysis of the structure of selected dance phrases. External recordings were thus used to corroborate how some actions unfolded in time, such as the interaction between the dance mentor and the apprentice, and the turn-taking and performance during improvisation. Conversations with team members further helped to corroborate the sequential unfolding of actions present in first-person insights. While the single-case autoethnographic design limits generalizability, its depth affords analytic transferability, yielding conceptual insights into the relational, rhythmic, and improvisational mechanisms that may inform comparable processes in other movement-learning contexts.

## 3 Case study: learning to improvise djembe dance

In this section, we investigate the nature of improvisation in djembe dance as it unfolds through the process of learning. We first outline the interaction system of djembe performance and the roles of repertoire, syntax, and mutual adjustment within it. As a second and critical step, we then examine attentional focus during the learning process and the role of memory—specifically, kinesthetic memory—in improvisation. We argue that, beyond recognizable repertorial elements and syntactic cues, the capacity to recall and mobilize dynamic movement parameters is vital to learning and performing djembe dance improvisation.

### 3.1 Music-dance interaction

In djembe performance, the relationship between dance and music is best understood as a dialogue—a multimodal expression and experience co-constituted through the interplay of musical and dance phrases—rather than as a unidirectional response of dance to music or vice versa (Djebbari, 2019; Polak, 2007, 2021). Djembe music-dance is characterized by a dynamic interplay between the rhythms produced by music and those generated through dance. Here, *rhythm* is understood as the organization of music or dance in time, structured through the regular alternation of accented and unaccented moments—such as up and down, tense and relaxed, long and short, or loud and soft (van Leeuwen, 2015). These contrasts produce rhythmic patterns in which accents are distinguished along diverse dimensions: in music, for instance, through timbre, pitch, duration, or volume; and in dance, through contrasts in force (e.g., peaks in kinetic energy), amplitude (e.g., reversal points in motion trajectories), or vividness of movement, among others.

The spatial configuration within which djembe music-dance interaction unfolds in social celebration contexts typically consists of a circular formation from which participants step into the center to improvise a movement sequence (Polak, 2021; in press). The initial section of each piece is devoted to group dancing known as *munumununin* (Bambara for “circling”), where dancers typically move counterclockwise around the dance ground in single file with both movement and the musical tempo remaining relatively subdued. In contrast, the second section of each piece is marked by an increased tempo and more vigorous dance movements, as individuals take turns performing brief solo sequences in rapid succession. This section is called *kelenkelenninbo* in Bambara, meaning “taking the floor one by one.” In some regional styles of djembe performance, such as that from Bamako, a particularly striking contrast emerges in the *golobali* phase (Bambara for “rushing” or “precipitation”), characterized by marked intensification and acceleration in both drumming and dancing, culminating in a climactic finale that may signal the end of an individual solo or of the piece as a whole (Polak, 2004, 2007).

In summary, dancers may enter the center of the circle to initiate a new dance phrase or to entrain with one already underway. When multiple solo dancers perform simultaneously with different phrases, the lead drummer must choose which one to engage with musically. These improvisational episodes are governed by several interrelated variables—foremost among them the piece of repertoire and the specific rhythmic patterns it affords. The following subsection is therefore dedicated to this dimension of djembe dance: its reliance on repertoire.

### 3.2 Repertoire

The performance of djembe music-dance is organized in repertoires of specific pieces (sometimes called “rhythms” in European but not West African languages), each identifiable by distinct drumming and dance patterns and associated with specific songs (Polak, 2012). For enculturated participants, performing djembe music-dance means recognizably realizing a specific one of those pieces available in the locally shared repertoire. These pieces also have names—for instance, “Maraka,” “Manjanin,” and “Wasulunka,” among others—and carry social and cultural meaning.

Tenin Malle, first author Marin-Bucio's dance mentor during fieldwork and a highly experienced djembe dancer with extensive practice in both social celebration and staged ballet contexts, notes: “Each musical piece is specific to one of Mali's ethnic groups. Repertoires are identified according to the ethnic origin of the sound and the dance” (2024). However, the djembe repertoire is not structured along the dimension of ethnicity alone but is multidimensional. For example, djembe pieces can also address identities at the level of age, region, status group, and hereditary profession, reflecting the complex relationship between djembe performance and society (Polak, 2012).

These pieces represent the highest hierarchical level of organization of the djembe repertoire. At the next level down, each piece consists of associated sound and movement patterns that form recognizable motifs and phrases. These are established through repeated practice and stabilized through social convention. While not usually named, they are still recognizable as spatiotemporal gestures. Tenin Malle, despite her extensive experience, could not determine exactly how many dance motifs exist for each repertoire, since new movements often surface in the dancing at social events in different regions. Nonetheless, Tenin is convinced that each piece has a set of specific movements associated with it, which she can identify:

I recognize a dance by the dancer's steps and without dance by the rhythm of the drummers. If a dancer dances Dansa, for example, without music, you can recognize the dance steps through their movements, (…) you could say that Maraka, Dansa, and Manjanin dance steps each have their own specific movements.

There is a paradox in djembe dance: the potential for inventing new motifs and phrases seems infinite, yet the repertoire remains recognizable and identifiable by the structures of each piece. No dancer can enumerate all possible motifs for a piece, but each new variation, to appear coherent, must still resonate with the shared expectations that mark it as belonging to the piece being performed. Elicitation interviews with Tenin and other professional dancers from Bamako, using video recordings of their performances, reveal flexible and nuanced approaches to how the repertoire is performed. Some dance phrases appear in multiple pieces, forming clusters of pieces that partially overlap while remaining distinct. For instance, certain Manjanin phrases may recur in some pieces but not others. The interpretation of these relationships can vary among dancers and is sometimes debated. For example, while all agree that Manjanin and Wasulunka must never mix, opinions differ on whether certain Manjanin phrases can appear in Maraka. Moreover, the identities of motifs, phrases, and pieces differ across local styles and evolve over time (Polak, 2012).

In summary, given a shared cultural context, enculturated djembe dancers know the music, while experienced drummers are expected to recognize the dance motifs. These repertorial units allow the participants to perform and interact. That is, just as the drummer's performance is framed by the musical phrases that define the piece in performance, the dancer draws from a movement vocabulary that—while not fixed—is shaped and conditioned by the structure and qualities of the repertoire being articulated. This interdependence informs the dancer's performance and delineates the range of possibilities for their participation. In the following subsection, we will turn to the question of how these repertorial units are sequentially developed.

### 3.3 Syntax, cues, and mutual adaptation

Improvisation in djembe dance differs in significant ways from improvisation in other dance genres. For instance, in some forms of contemporary dance, what counts as prototypical for improvisatory processes is the constant innovation of movements—that is, drawing upon the movement mechanisms learned by the dancer to then deconstruct and regenerate them as flexible patterns. The attention need not be strictly linked to music, since dancers can engage with more abstract representations of rhythm, such as somatic indicators,^11^ and use the conceptual imagination^12^ to improvise dance. What counts in contact improvisation, for example, is the flow between partners, which depends on clear mechanisms for transitioning through lifts, falls, and bodily interactions. Yet, these structures are not strictly repertorial; rather, they are continually transformed in response to the emergent dynamics of each encounter.

By contrast, djembe dance operates within a different improvisational logic, one that is based on the mobilization of repertorial units and an entangled dialogic interaction with the drummer. Importantly, in djembe performance, both drummers and dancers exchange cues to negotiate the development of the music-dance improvisation. Comparable forms of repertorially structured improvisation have also been observed in other African music-dance traditions, though each articulates this logic in its own culturally specific way (see Carlozzo, 2019; Gerstin, 1998). Within this interaction, the attunement between the dancer and the lead drummer is mediated through two mechanisms that we term *rhythmic offers* and *calls*.

By rhythmic offers, we mean danced proposals that invite a musical response, such as an adjustment in tempo or rhythm. Interaction through rhythmic offers arises when a dancer enacts a movement motif and the lead drummer adjusts so as to accompany it musically in a congruent way.^13^ Calls, by contrast, refer to explicit signals given by the lead drummer to the dancer to announce formal changes. They can cue the initiation^14^ or conclusion^15^ of the dance or invite the dancer to propose a new dance motif.^16^ These moments—their occurrence, their duration, and the ensemble's responses to them—are not fixed but instead coproduced and negotiated as the performance unfolds. For example, exactly when the lead drummer plays the call to end the dance is contingent on the dancers' prior choices—in some situations, that is, the drummer should do so only after the performers have transitioned into the *golobali* and the dance has reached the fastest tempo possible for the current dancer. The increase in tempo emerges through mutual adjustment—dancers quicken their movement as drummers accelerate the beat.

This process is characterized by the dancer's initiation, alteration, or recombination of motifs drawn from a style-specific vocabulary. The critical moment when a dancer steps into the circle or shifts the structure of their movement is a situated act that emerges at the intersection of that vocabulary and real-time dialogic entrainment, wherein only certain options are both viable and stylistically intelligible at that moment. This act may sometimes appear fixed or choreographed from the outside, yet it is fundamentally improvisational because it arises from a real-time, embodied response to the musical cues and interaction dynamics. Most importantly, the dancer must attune to the repertoire and rhythm cued by the drummer, the spatial and interactional conditions of the circle (proximity, turn-taking, audience energy), and their own embodied readiness (breath, fatigue, intention). At the same time, the drummers' adjustment to the dancer is equally critical, making the djembe performance a process of bidirectional adaptation: a music-dance performance.

In summary, djembe dance improvisation prioritizes interaction with the lead drummer based on repertorial units and a shared understanding of syntactic cues such as rhythmic offers and calls. These communicative tools are established through social practice, linking dance units (motifs and phrases) with musical form units. These tools support the shaping of a linear yet flexible and responsive flow of real-time performance compositions.

### 3.4 Pedagogy and the learning process

Given the ways in which djembe improvisation takes place within an interaction system wherein recognizable and identifiable units of repertoire are unfolded and sequenced over time, we now turn to the question of how a newcomer to this cultural system might acquire the necessary improvisational skills.

**Figure 3:**
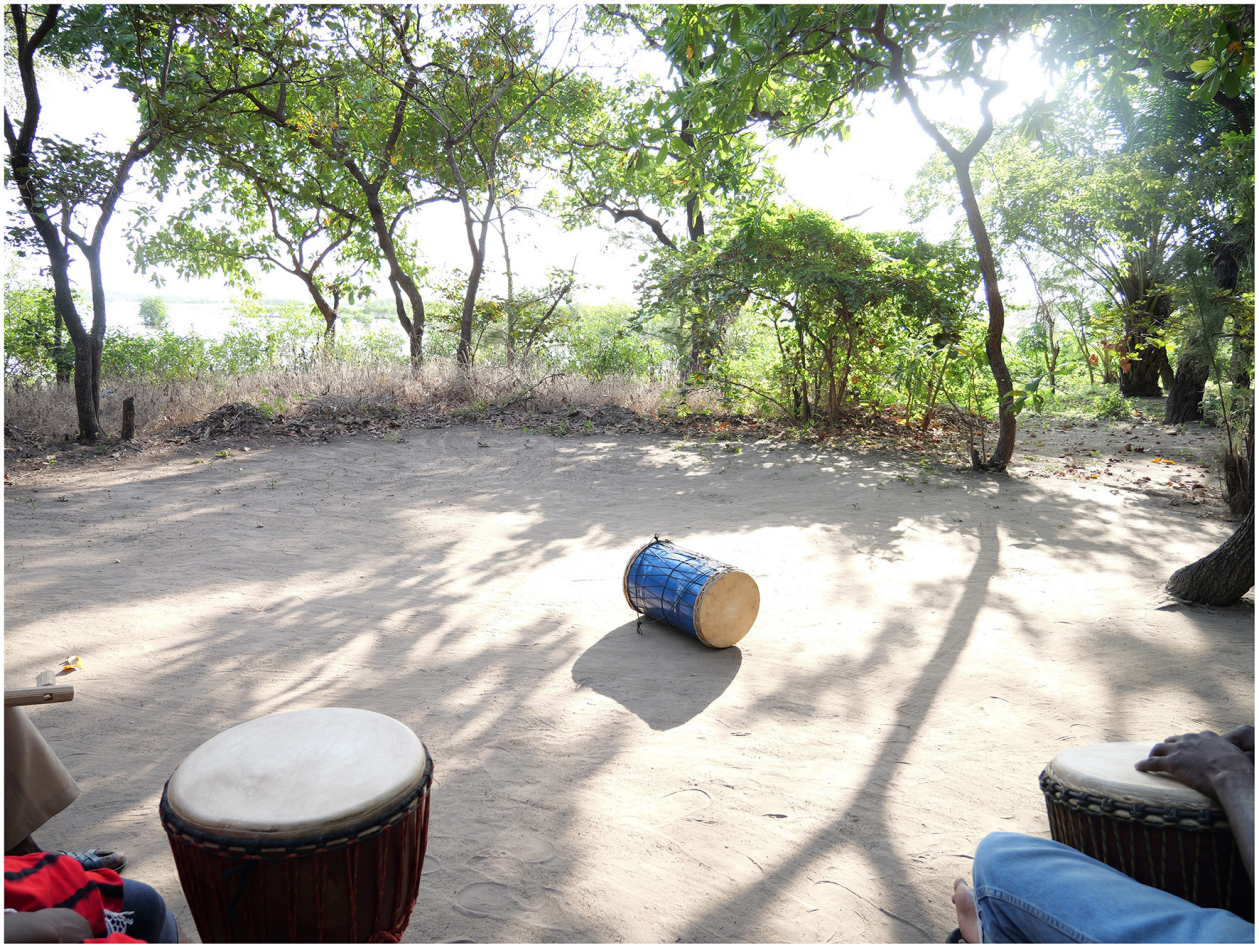
Outdoor space where the dance sessions took place (Kafountine, Senegal, October 2024). Photo: Diego Marin-Bucio.

Within the fieldwork's daily routine, when the morning session began, my focus was on dance learning in an outdoor clearing surrounded by trees, bushes, gardens, and an estuary waterfront (see Figure 3). The drummers sat on chairs facing the center of the space, while the dancers were barefoot in contact with the soil. The session started with a brief warm-up, moving joints and stretching limbs in attunement with the drumming. In the first few weeks, my attention was primarily focused on mimicking my teacher's movements. For each new movement, my acquisition of it required two phases: *on the spot* and *locomotor*. Her pedagogical approach to the first phase was based mostly on demonstrating one phrase at a time. The dance movements were presented to me on the spot, face-to-face, and if this mirror perspective grew overly complicated, I would stand behind her as well. She typically introduced dance phrases while the drumming was already underway, and only when I encountered difficulties would she ask the musicians to pause. Practicing in silence rarely happened but was especially helpful for learning more complex phrases, as it allowed me to concentrate on specific elements such as weight placement and the spatial trajectory of my movements. In these cases, once I succeeded in reproducing the sequence, we would reintegrate it with live drumming. In the second phase, which involved moving around the circle, I was required to translocate my body while performing the movements. This locomotive adaptation of the phrases sometimes introduced new challenges, such as converting stomps into steps to advance along the circle's trajectory. Some motifs, even when practiced in place, already entailed displacement—for example, a Manjanin motif requiring four steps forward followed by four backward. To move effectively around the circle, I modified the motif by lengthening the forward steps and shortening the backward ones, enabling smooth translocation in a frontal, counterclockwise direction.

Although my learning took place in a pedagogical setting, the principle of observation and imitation as the primary mode of acquiring djembe dance remained central. This mechanism of learning through attentive observation is also prevalent in traditional contexts. Polak and Doumbia (2021) quote a senior Malian farmer affirming this practice:

No one taught me how to dance. I just have to observe how you move, the rest is up to me, it's up to my mind. If I just watched you dance, that's all. A dancer first sees someone's movement, then he/she has a picture of the dance in his/her mind, and then he/she is able to perform it, in practice. There is no teaching, no rehearsal. All you need is to be attentive, and then you put it into practice yourself. [Keita, 2019]

This mode of learning was also described by my dance mentor:

Because of my young age, I quickly picked up the dance steps by observation, without making any direct movements (…) [Years later] there was a cultural space in Kayes called AMICAL occupied by the region's dance troupe—the old drummer's name was Sidi Traore (Sididjan). The troupe was preparing for the interdistrict competition (…) I danced exactly like my friend, in perfect harmony. At the end, everyone was surprised and amazed by the quality of my dancing, because I was new. I declared that my mother is a dancer, and I'd watched her dance a lot.

Throughout this process, the role of the drummers was essential—not only as accompanists but as active agents in the learning environment. As Cruz Banks (2023, pp. 299–300) notes, djembe drummers “provide a sensorial warm-up for heightening dancers' music discernment,” display their skills in their musical listening and alignment, and support the development of a choreographic practice that aims to “make good music.” These drummers' contribution to learning goes beyond maintaining tempo; it shapes the dancer's rhythmic perception, attentional focus, and compositional choices throughout the process.

**Figure 4:**
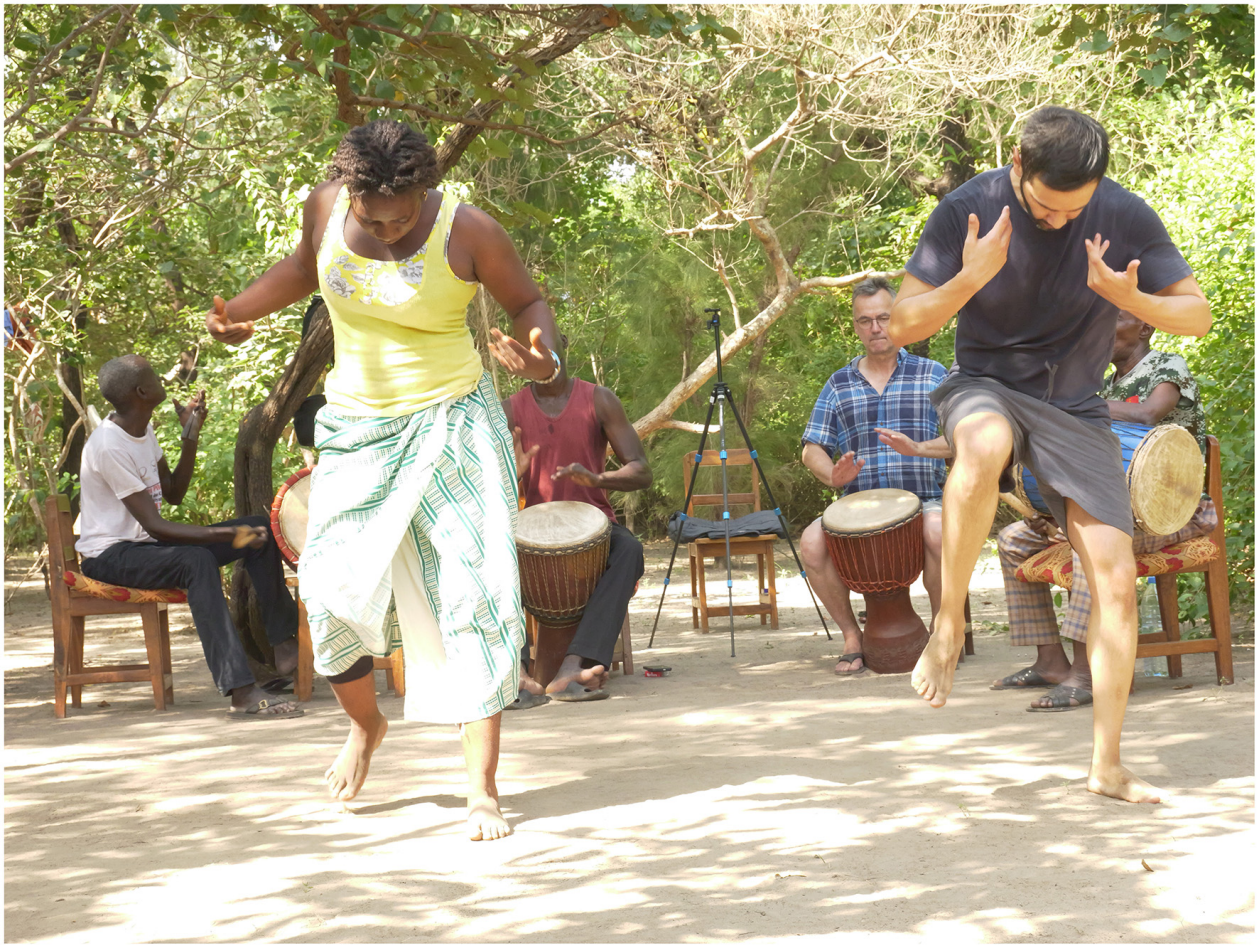
Tenin Malle leading a dance session with first author Marin-Bucio as an apprentice. The image illustrates the process of learning a movement of the Dansa rhythm. The four drummers are (from left to right) Vieux Camara, Ba Issa Traole, Rainer Polak, and Madou Diakité (Kafountine, Senegal, October 2024). Photo: Noumouké Doumbia.

Although I was physically engaged in performing the steps, I initially depended on my mentor's demonstration to maintain my rhythm and form (see Figure 4). When she paused and I had to rely solely on my own recall, I could manage simple or slow movements by repeating them in a loop, but sustaining more complex motifs without her visual cues often became a challenge.

Over time, however, I became able to anchor my movement in how I had internalized the structure of the dance, relying increasingly on my embodied experience. This shift freed my attention to engage with subtler dimensions of performance: the trajectories traced through space, the modulation of muscle tone, the alignment of posture, and the fine articulations of the joints. Yet even as I executed the steps with accuracy in timing and shape, I became aware that my movements carried an aesthetic imprint from my prior training in Western modern and contemporary dance. The way I lifted my arms, the pathways I traced, the density of the muscular effort, the placement of my wrists and fingers, the carriage of the spine, the tilt of the head, even the way I inhabited verticality—-my upright body posture while standing, walking, and dancing—all revealed traces of another body aesthetics and movement lineage. Slowly, I had to inhibit these ingrained mechanisms to approximate the effortless vitality I observed in my teacher. What she embodied was not simply clear form but also a certain quality of flow—light yet strong, released yet precise, joyful yet grounded. The subtle tilt of the hips, the gentle bend of the knees, the rhythm carried through the head, the release of tension at the fingertips—all these aesthetic inflections were integral to the djembe dance. They were not handed to me explicitly, through gestural indication or verbal instruction, but remained something I had to decipher and inhabit for myself. This became a second central task of my learning: not only to enlarge *what* I was able to do but also to transform *how* I did it, until the movement resembled ever more the performance style of my mentor and indicated my increasing proximity to this genre-specific movement aesthetic.

Over the course of a month, I sufficiently internalized my motor control such that my attention could shift toward syntactic cues such as the lead drummer's calls and the rhythmic offers of fellow dancers. This attunement evolved concurrently, though at different rates, rather than in a strictly sequential manner. By the second week of training, I was already participating in improvisational exchanges, engaging in the djembe performance as an interactive agent rather than merely following my instructor's lead.

This process of developing from self-awareness toward responsive interaction resonates with West African perspectives upon embodied consciousness, such as the notion of the body as a site of knowing and being that is found among members of the Anlo-Ewe ethnicity from southern Ghana (Geurts, 2005, p. 168). This notion begins with *seselelame* (feeling in the body), moves through *gomesese* (understanding), and leads to *sidzedzenu* (recognition). From this perspective, a dancer's growing embodiment of movement reflects a deepening integration of sensory perception, cultural understanding, and situational recognition, which together constitute a holistic form of conscious engagement in performance. In my experience, then, performative communication and interaction between dancer and musician was the last stage of learning djembe dance improvisation because my attention had to transcend my performance and embrace stimuli from and interaction with my co-performers, including both drummers and fellow dancers.

### 3.5 The moment of decision-making in djembe dance improvisation

Improvisational interaction in dance requires a substantial foundation of embodied knowledge that can only be developed through exhaustive and sustained embodied learning. Skill acquisition for expert performance in specialized tasks, after all, depends on long-term, deliberate practice (Ericsson et al., 1993; Ingold, 2000). As outlined in the previous subsection, before dancers can improvise effectively, they must internalize a repertoire of movement vocabulary, rhythms, and response patterns, allowing spontaneous action to emerge from deeply ingrained structures. In this subsection, we investigate how these conditions are brought into play in the spontaneous decision-making that improvisation demands.

Research employing computational corpus studies and neuroscience methodologies, respectively, has demonstrated that creative improvisation in music (Beaty et al., 2022) and dance (Yang et al., 2024) often begins with the retrieval of well-rehearsed, easily accessible sequences, which are then elaborated into more complex and original material. As has been reported by Merseal et al. (2023), improvisers tend to rely on structured learned patterns when generating novel material, supporting the idea that improvisation is guided by deeply internalized knowledge rather than arbitrary choices.

**Figure 5:**
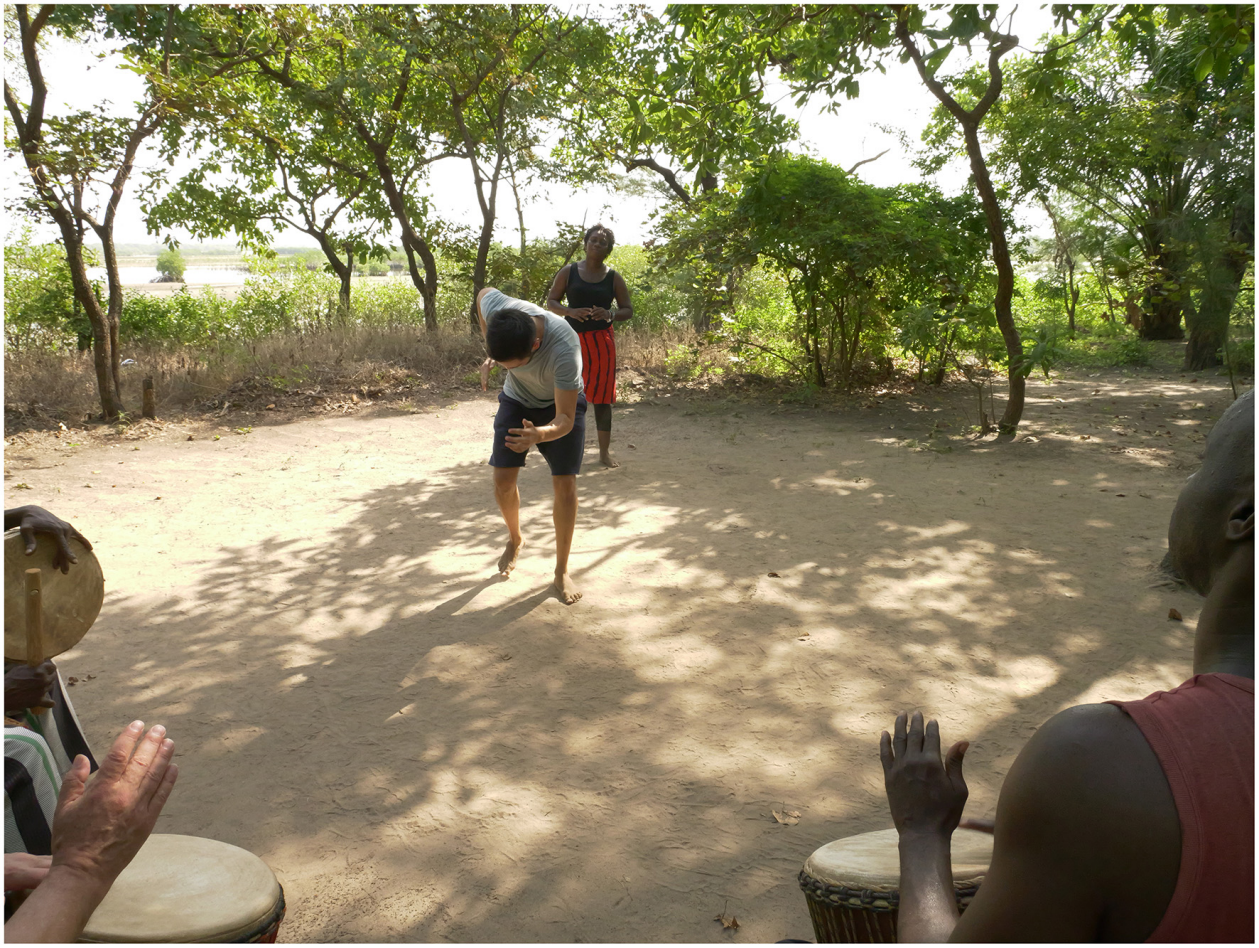
Tenin Malle supervising my improvisation performance within the rhythm Wasulunka. Ba Issa, the djembe lead drummer (right side), keenly observes and interacts with the dance improvisation (Kafountine, Senegal, October 2024). Photo: Noumouké Doumbia.

In djembe dance, improvisation is constrained by the rhythmic framework of each piece and requires the use of specific motifs and phrases embedded in the cultural logic of the dance. For example, if we were performing Maraka, I was using only the appropriate movements for this piece. Therefore, my dance improvisation was tuned to how I had internalized the repertoire as I had learned it during my fieldwork. Even without a complete historical or cultural context, my improvisation was already beginning to be guided by the vocabulary my mentor affirmed as appropriate within the tradition (see Figure 5).

However, while my dance vocabulary was shaped by the social conventions of repertoire, the process of selecting specific movements during improvisation also depended on situational and individual factors. During an interview, I asked my dance mentor what influenced her decision to choose a specific movement when improvising, and she responded: “When I listen carefully to the sound the drummers are playing, in my head I prepare the dance steps according to the beat of the music and improvise” (Malle, 2024). Her answer highlights the importance of music and dance interaction and their temporal coordination. However, it also presupposes an internalized repertoire of culturally informed movement vocabulary and aesthetic values that guides her improvisation—elements so embodied that they may not be consciously articulated.

In djembe dance, the moment of improvisation starts with the dancer's intention to enter the circle and initiate or alter a phrase or motif. This often requires immediate adaptation on the dancer's part. The dancer may enter the center with a particular phrase in mind, only to find that the preceding dancer's movements have shifted the musical phrase and/or the tempo played by the drummers, rendering the planned dance phrase incompatible with the current rhythmic flow. During our fieldwork, I encountered this firsthand myself. In my initial attempts at improvisation, I often noticed an unexpected shift in the tempo just before my turn to enter the circle—a moment that disrupted my prepared phrase and forced me to adapt in real time. These improvisation sessions were video recorded, which allowed me to reflect on each one afterward. This helped me track how my awareness, responses, and movement choices changed over time, as my fieldnotes indicate:

Today I was very conscious of a moment in which I had planned to do a move, but I felt the steps that Tenin (my dance mentor) was doing were changing the state of the musical rhythm, and that made me feel unsure if the steps I wanted to do were going to fit well. And when my turn was coming up as she was exiting the center, in my head I was thinking, “do this one . . . no, better this one.” In the end, I'm not entirely sure how I chose; I just danced, and it worked (Fieldnote, November 2024).

In this example, the musical rhythm and the kinetic imprint of the previous dancer (Tenin) functioned as context, shaping the decision-making of the subsequent dancer (Diego). I adjusted my intuitive response to cohere with both the ongoing rhythmic stimuli and those that had been previously established. This example illustrates how the moment of decision-making attunes to the evolving state of the performance, whereby the agency of the other participants can alter or shape the dancer's selection of motifs and performance qualities such as tempo.

The available dance movements also vary according to the different sections of the performance, each of which unfolds in a distinct tempo range. For instance, the motifs that might be used during the slow group-dance section in each piece, the aforementioned *munumununin*, may differ from the ones executed in the faster *kelenkelenninbo* section. In the *munumununin*, dancers typically move in a counterclockwise circle, with both movement and musical tempo remaining relatively subdued. Tempo increases when dancers enter the center to improvise,^17^ but the most substantial contrast comes during the third section, *golobali*, which is characterized by a significant intensification and acceleration of both drumming and dancing. This final climactic section is realized by both the drummer and the dancer using specific motif types designed to work well at the highest tempo range.^18^

Beyond the temporal parameters, each of these sections also has spatiotemporal and energetic implications. For example, in the pedagogy and learning section, we described the use of circular translocation in the dance ground, which is typical of the *munumunnin* section. In the following subsection, we are going to describe its energetic movement aspects.

### 3.6 The unfolding of kinesthetic memory: beyond repertoire and syntax

As discussed above, kinesthetic memory is the process of accumulating embodied knowledge through repeated sensations and actions, enabling dancers to draw on past bodily experiences that are mobilized to shape new movement in the moment (Sheets-Johnstone, 2011). I experienced the critical importance of kinesthetic memory when trying to recall a dance phrase that I learned during fieldwork. What struck me was how the act of remembering was inseparable from doing: I could not access the sequence as an abstract idea but only by reenacting it with my body or, later, by projecting the movement internally. The following fieldnote captures this insight:

At the beginning, the only way I could remember the movement was by physically performing it. As soon as I initiated the first step, the next one would emerge instinctively, until the full sequence unfolded. Eventually, I became able to recall the movements without moving, but even then, I would mentally dance it—I could imagine the front of my body from my own visual perspective and re-experience the bodily sensations tied to the movements. It feels as if a light illuminates the next step once the previous one is finished. In both cases, the dance revealed itself over time, as if each sensed movement called forth the next (Fieldnote by the author, November 2024).

This experiential account resonates with Luria's (1973, p. 176) concept of *kinetic melodies*, in which a single motor impulse triggers a patterned chain of movements.^19^ Applied to djembe improvisation, these mechanisms illuminate how readiness in body and mind enables partially prestructured yet adaptable responses. Even when a dance is memorized, its recall depends on embodied enactment—whether physically performed or internally imagined. This embodied account aligns with Sheets-Johnstone's perspective:

Movement and perception are seamlessly interwoven; there is no “mind-doing” that is separate from a “body-doing.” My movement is thus not the result of a mental process that exists prior to, and is distinguishable from, a physical process in which it eventuates, nor does my movement involve no thinking at all (2011, p. 422).

While Sheets-Johnstone's distinction between *thinking in movement* (movement as a primary cognitive process) and *thoughts of movement* (intermittent reflective moments) is often exemplified through contemporary dance improvisation, the enactment of embodied memories is fundamental and plays out differently in different dance genres. Both djembe and contemporary dance improvisation draw on kinesthetic memory; what differs, in my experience, is the mechanism by which it is mobilized and the constraints that circumscribe viable choices. Some techniques of contemporary dance improvisation, such as contact improvisation or Gaga (Katan, 2016; Paxton, 1975), often engage and play with aspects of kinesthetic memory that do not rely on recognizable, identifiable motifs but instead on qualities of movement.^20^ For example, a dancer might not recall a specific gesture but will recall the feeling of suspension, the texture of vibrating limbs, or the momentum of a twisting spiral—which then informs their improvisational choices. In this context, dancers navigate felt states as raw material for generating new movements. In contrast, in djembe dance improvisation, dancers use their kinesthetic memory to fluidly execute movement via well-practiced motor patterns. Such kinetic chains in the context of djembe dance are drawn from a vocabulary of recognizable motifs and phrases. Once initiated, the dance movements tend to unfold with both procedural fluency and cultural coherence, allowing the dancer to remain responsive to the live drumming while sustaining the social-musical logic of the form. In both genres, the dancers reactivate a shared cultural framework that allows them to improvise within their respective cultural context. They do so through different epistemologies, however: in djembe dance, kinesthetic memory is mobilized as genre-specific repertorial units, whereas in some techniques of contemporary dance, it emerges through sensorial and dynamic processes shaped by social conventions of attending to and transforming movement.

**Figure 6:**
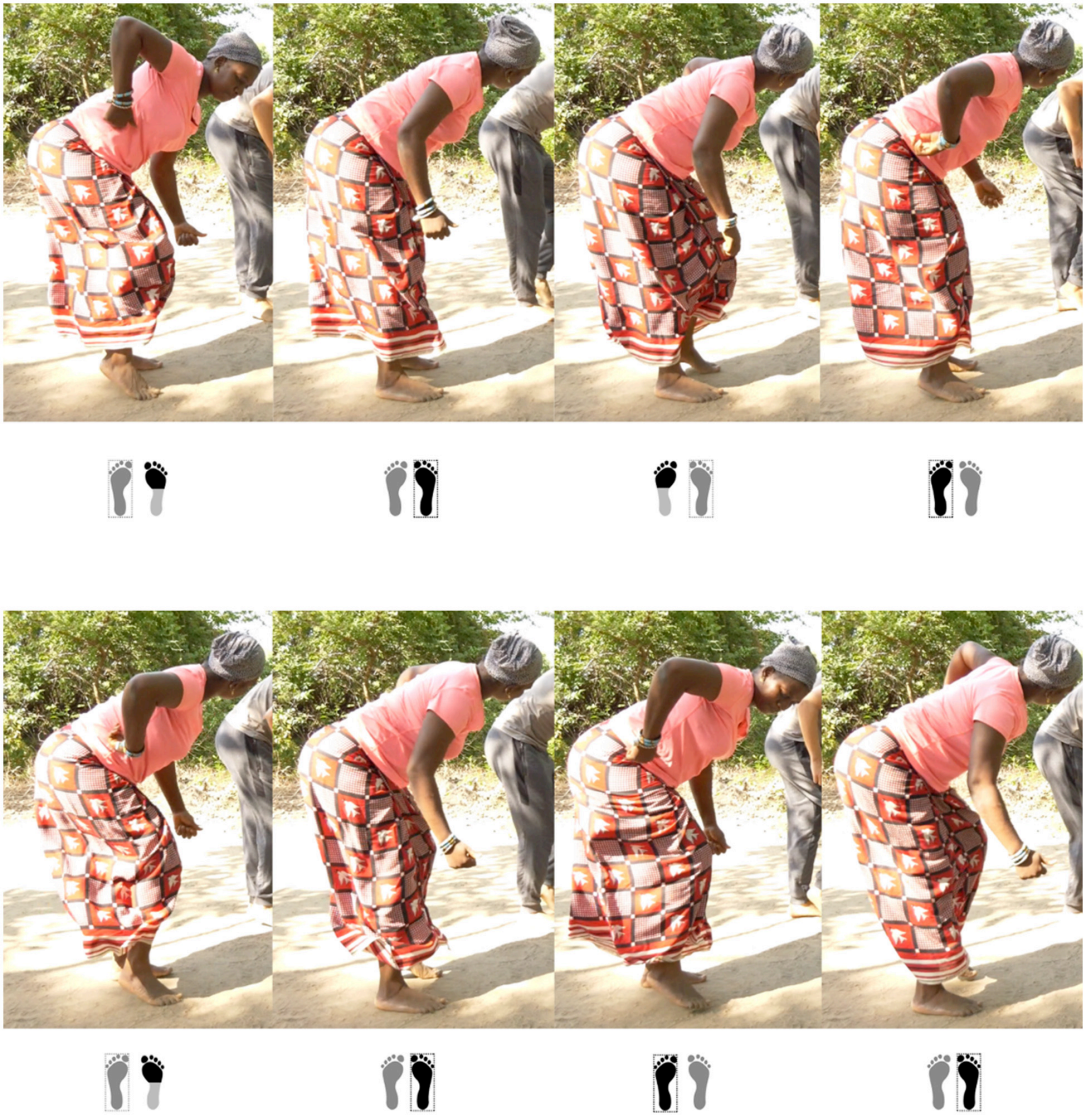
Video still showing dancer Tenin Malle performing a dance motif from the Wasulunka djembe rhythm. Within the Bamako style of djembe dance, the piece Wasulunka, as well as other pieces from the region Wasulun (Wassoulou), revels in rapid footwork. The footprints represent contact with the ground. Feet framed individually represent the foot that holds the body weight. Black in the footprint marks the part of the foot in dynamic contact with the ground. See the video example of this dance motif from Wasulunka rhythm: https://zenodo.org/records/16678699 (Kafountine, Senegal, November, 2024).

**Figure 7:**
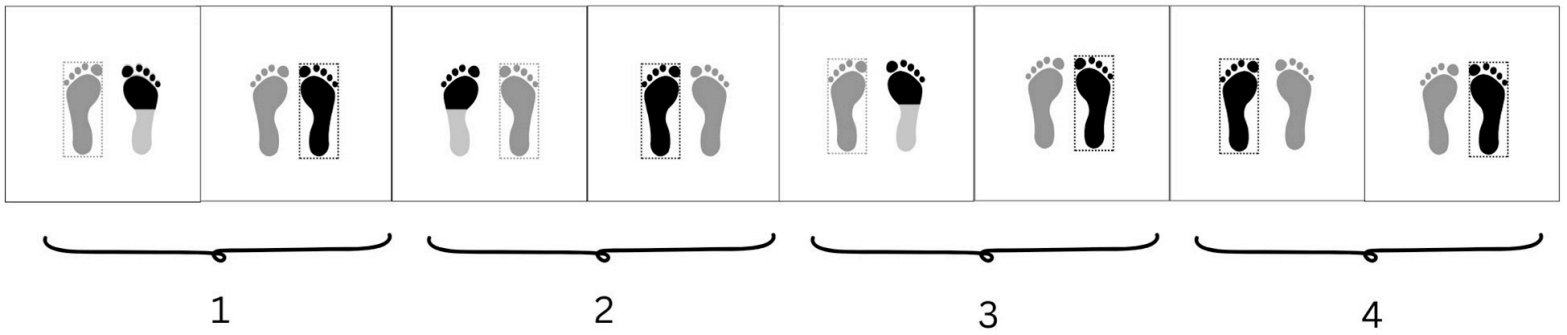
Representation of weight distribution across a four-beat cycle of a dance motif from the Wasulunka djembe rhythm as learned during fieldwork.

**Figure 8:**
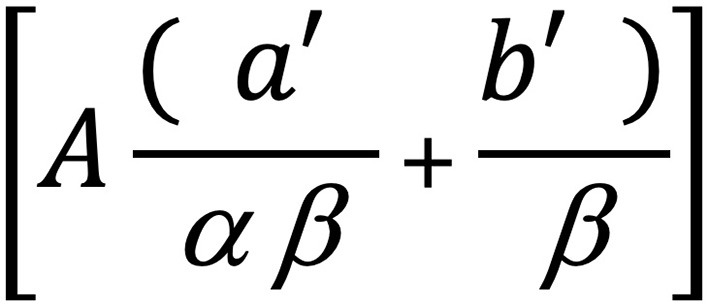
Structural components of the motif analyzed from the Wasulunka djembe repertoire. This motif [A] is composed of two motif elements ([a′] and [b′]), each of which can be further subdivided into two cells ([α ] and [ β ]). It alternates body weight between the right (R) and left (L) foot in a four-beat cycle: one cycle begins with the right foot (R-L-R-L-R), then repeats with the left (L-R-L-R-L).

**Figure 9:**
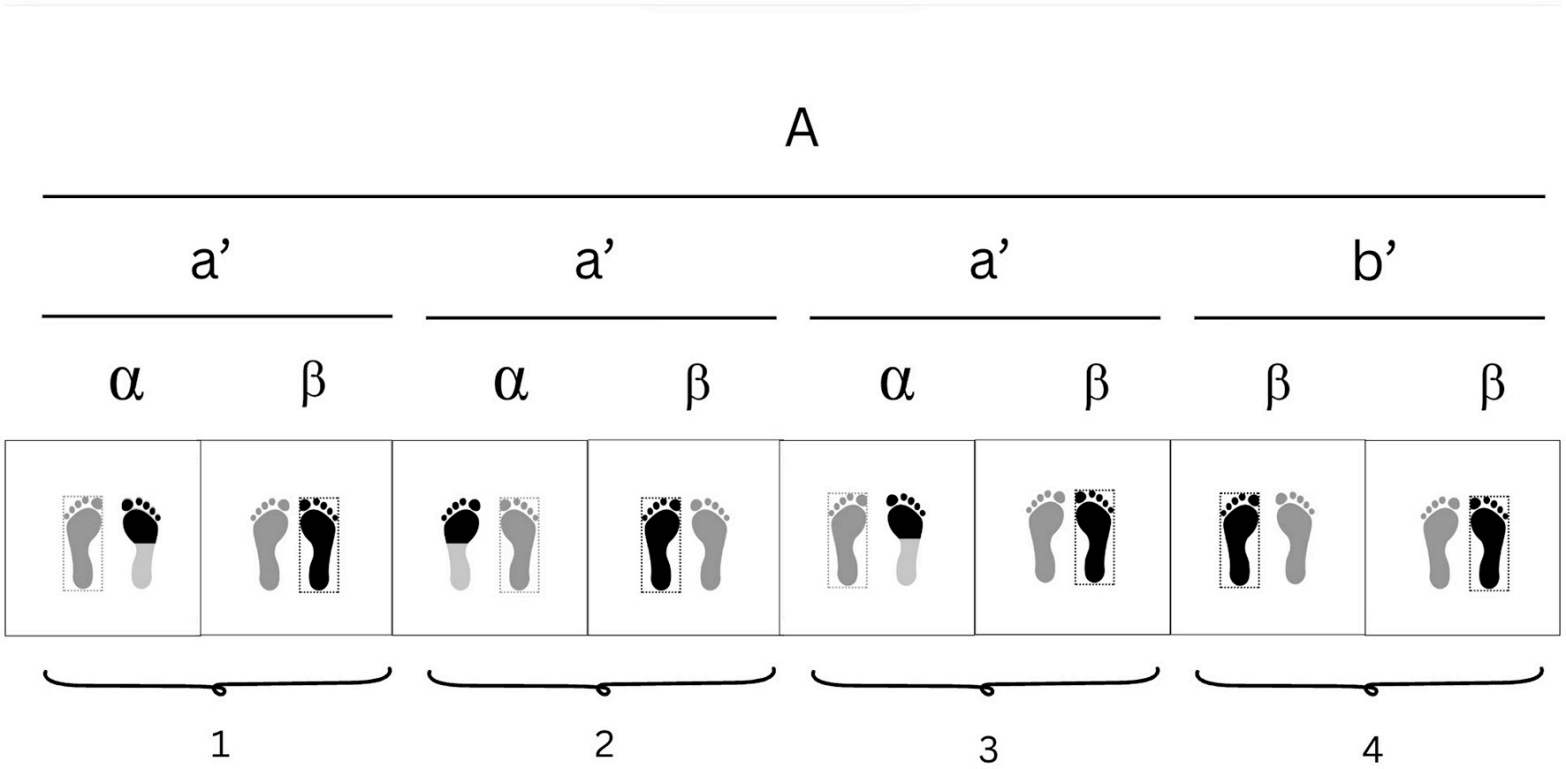
Integral form of the analyzed motif (see Figure 8) from the djembe piece Wasulunka. The first three weight shifts occur across three beats utilizing element [a′], while the fourth and fifth weight shifts encompass element [b′], which represents two rapid changes compressed into one beat. Motif element [a′] subdivides into two smaller cells: [α], a metatarsal tap against the floor, and [ β ], a placement of the sole. Motif element [b′] mobilizes motif cell β twice. The polykinetic nature of the motif is further emphasized by the arms, which alternately bend and extend at the elbows so that the extended arm corresponds to the placement of the sole. The movement is performed with slightly bent knees, a forward inclination of the torso, and the head facing downward.

During my learning of djembe dance, both of these modes of accessing and mobilizing kinesthetic memory surfaced in fundamental ways—sometimes clashing with my performance intentions, other times enabling the execution of specific movement mechanisms. As mentioned earlier, I occasionally had to progressively remove the automatic movement qualities I was unconsciously transferring from my contemporary dance background to my new djembe dance movements. However, there were other moments when such kinesthetic memories were essential to my learning of new movement mechanisms and dynamics. For instance, when learning the piece Wasulunka, I encountered a motif (Figure 6) involving rapid footwork that combined dynamic snaps against the floor with shifting weight (Figure 7). What was most striking to me was not its structural complexity (Figures 8, 9) but its dynamic principle and underlying kinetic intention. This dance motif depends on a rebound action: precision and speed emerge not from stomping or pressing down into the ground but, on the contrary, from *rejecting* the floor upon contact. I recognized this kinetic logic by drawing on my experience with the “prance”^21^ in modern dance, where the supporting foot does not simply push into the ground but actively propels the body weight, producing a quick upward rebound that enables lightness, clarity, and velocity. This mechanism was pivotal in allowing me to reach the speed required by this Wasulunka motif, whose structure can only be performed when force is applied in the way described above.

A motif, after all, is not only a sequence of positions in space, and performing the spatiotemporal trajectory of the Wasulunka example alone was not enough—it had to be executed at the appropriate speed, which required the upward rebound as well as a set of qualitative movement dynamics that lend the motif its character. Only the combination of these factors allowed me to perform the Wasulunka motif with its inherent aesthetic and rhythmic intention, and what enabled me to access this kinetic principle, again, was recalling the logic of prances from my earlier training in modern dance. Of course, this is only one possible route: dancers whose kinesthetic memories were shaped through other movement systems (dance genres, martial arts, sports, physical labor, etc.) certainly can arrive at the same mechanism through different resources. Needless to say, djembe dancers can come to it directly from within the djembe tradition itself.

## 4 Discussion

### 4.1 Improvisation as the mobilization of kinesthetic memory

This study examined the role of kinesthetic memory in improvisation through an autophenomenological approach realized in the context of short-term ethnographic fieldwork on djembe dance, complemented by interviews and focus groups with experienced practitioners and last author Polak's long-term ethnographic and collaborative experience with the same practitioners. The analysis starts from the assumption that memory is essential as a resource for dance improvisation. Findings show that kinesthetic memories concern repositories of spatiotemporal patterns (genre-specific motifs and phrases) but also include dynamic movement qualities. Formed within cultural frameworks that shape their enactment, these memories function to link individual embodiment, social interaction, and cultural framework.

In djembe dance, these resources correspond to repertorial units recognizable to enculturated practitioners within specific stylistic, historical, and situational contexts. While repertorial grounding characterizes many dance genres, some contemporary forms—such as contact improvisation, release, or Gaga—do so less prominently (George, 2020; Katan, 2016; Paxton, 1975). By contrasting the mobilization of both repertorial units and movement qualities from within kinesthetic memory, our research highlights how dancers from different traditions recall and shape movement through distinct yet complementary modalities of embodiment. Both aspects arise through the embodied process of thinking in movement and are situated within specific cultural and social contexts.

This complementarity shows that, on the one hand, improvisation, even in seemingly abstract forms like contemporary dance, remains structured, grounded in social conventions of sensing, attending to, and transforming movement. On the other hand, even in allegedly traditional forms like djembe dance, improvisation does not operate only by mobilizing repertorial units; it also involves the dynamic modulation of kinesthetic qualities embedded in memory. Take, for example, the Wasulunka motif we described in the previous subsection: although it could theoretically be executed at a slower pace, its aesthetic essence would be lost outside the fast tempo that activates its distinctive rebound quality and realizes its aesthetic intention. This example illustrates how far improvisation in djembe dance requires mobilizing not only the *what* but also the *how* to perform a specific movement pattern.

These findings align with the concept of procedural memory—the system enabling skilled action without conscious deliberation (Sanchez et al., 2010)—but emphasize that such memory is genre specific and culturally constituted. How movement is remembered and recognized depends on the frameworks in which it is learned and performed. Similarly, music performance (Polak et al., 2026) and public behavior more broadly unfold within particular social contexts (Bourdieu, 1984; Goffman, 1974). Hence, improvisation cannot be understood apart from the situated learning and performance processes through which kinesthetic memories are cultivated.

The study thus advances the understanding of dance improvisation in two respects. First, we emphasize the importance of kinesthetic memory and its contingency on processes of social learning and cultural framing. Second, we show that improvisation extends beyond the recombination of fixed patterns to encompass qualitative, relational, and context-specific dimensions. The study demonstrates how embodied memory is reconfigured through rhythmic dialogue and responsive adaptation, offering a transferable framework for understanding how dancers internalize and translate unfamiliar movement systems in the course of learning processes. By situating improvisation within a dynamic ecology of individual perception, social interaction, and cultural exchange, we bridge phenomenological and ethnographic perspectives, emphasizing the situated and relational nature of intercultural embodied learning.

### 4.2 Thinking in movement

As the above makes clear, improvisation in dance is not simply about making movement *fit* but about making movement *work*—in alignment with repertoire, kinetic possibilities, and the expressive logic of the form. This means remaining rhythmically responsive while honoring both the biomechanical constraints that govern movement dynamics and the cultural integrity that defines when, what, how, and at what tempo a dance motif can convincingly come to life. However, while this perspective emphasizes the cultural and social frameworks that shape djembe improvisation, it is also necessary to consider how the act of improvising is “lived” by the dancer in the moment. Phenomenological accounts of dance experience shed light on this dimension, showing that improvisation arises not from detached planning but from the ongoing flow of movement itself. In this sense, it is not a matter of mentally projecting possibilities in advance but of thinking in movement, the aforementioned continuous flow in which perception and action unfold together. In a contemporary dance improvisation, I experience this most clearly when each movement seems to generate the next without my needing to plan ahead. A slight twist in my torso invites my arm to arc outward; that arc pulls my weight off-center, and the imbalance draws me into a fall. As I fall, my feet reorganize beneath me, catching the momentum and redirecting it into a turn. I am not selecting these actions in advance—they arise because I am attending to what the movement is already doing: the pull of gravity, the texture of the floor under my feet, the stretch in my ribs, the timing of my breath. These sensations register immediately as invitations, not as ideas I deliberate upon. In this way, the improvisation unfolds as a continuous loop of sensing and acting, where perception and movement coemerge. Within this flow, thoughts of movement may surface as images of particular gestures, yet they emerge from the movement itself rather than from deliberation.

Thoughts of movement arise within the very act of dancing—culturally and sensorimotorly contingent, they appear and dissolve according to their contextual potential. During improvisation, such thoughts often diverge from the movements performed. For instance, in the context of contemporary dance, while dancing a floor sequence I might imagine jumping, yet as I improvise and eventually rise to standing, shifting sensations and intentions transform the moment itself, and the impulse to jump has already vanished. In the context of djembe dance, the earlier example presented in the case study—when the phrase I had in mind for entering the circle vanished as another dancer's rhythm shifted the groove—shows how such movement images may surface but dissolve in the ongoing dynamics of dancing, continually reshaped by the body's attunement to change. Thus, dance improvisation can be understood as a kinetic intelligence that both shapes and is shaped by the dynamic patterns within which it unfolds. It is in this simultaneity of shaping that both modes coexist in real time, as thoughts of movement emerge as “spin-offs of thinking in movement, rather than the result of an ongoing process of thinking in images while moving or the result of any deliberative thinking” (Sheets-Johnstone, 2011, p. 423).

To conclude, our account of learning djembe dance in fieldwork, together with practitioners' insights, demonstrates how individual embodied histories, including training, experience, and everyday movement habits, shape both thoughts of, and thinking in, movement—that is, how dance is learned, remembered, and performed. This highlights how culturally specific procedural memories condition the development of improvisatory resources and skills. Through learning, dancers accumulate vocabularies that resurface in improvisation, defining both the scope and the limits of creative action. Taken together, these insights position kinesthetic memory as a vital dimension of improvisation—one shaped by cultural frame, social situation, and biographical experience. Improvisation thus emerges not as free invention but as a dynamic negotiation between embodied recall and cultural expectation, between personal movement histories and collective frameworks that render dance legible and likable. Emphasizing the role of qualitative kinesthetic memory alongside patterned motor units offers a more comprehensive account of the mechanisms through which movement is perceived, shaped, and generated in real time.

## Data Availability

The datasets presented in this article are not readily available because the raw audiovisual and observational materials require extensive contextual knowledge to be meaningfully interpreted and may lead to misrepresentation without researcher-mediated explanation. Requests to access the datasets should be directed to Diego Marín, dabucio@uio.no.

## Footnotes

1 A note on the usage of first-person singular and plural: given the partly autoethnographic orientation of this multi-authored paper, we use the first-person singular when expressing lead author Marin-Bucio's perspective and switch to the plural when representing the author team's perspective.

2 Contemporary dance resists aesthetic fixation (Pouillaude, 2004). Yet, in academic contexts it is often associated with hybrid vocabularies that merge release, contact, and modern techniques with non-Western and street dance forms (Kwan, 2017). Its practices derive from dance theorist Rudolf Laban's ideal of movement as a language of self-realization and creative expression (Bakka, 2020, p. 2), reflecting plural sociocultural contexts and versatile modes of training.

3 A similar perspective on dance improvisation as a form of unconscious competence is put forward by Ribeiro and Fonseca (2011).

4 In our analysis, we use the term *motif* to denote the smallest movement unit that conveys meaning for experienced performers and audiences within a given dance system (Giurchescu and Kröschlová, 2007, p. 28).

5 A *phrase* comprises one or several motifs and exceeds the mere aggregation of these elements; it operates at a higher qualitative level, possessing a distinct and recognizable identity (Giurchescu and Kröschlová, 2007, p. 30).

6 The presence of preexisting elements does not mean exclusively literal reproduction. In some contexts of contemporary dance, improvisation depends on the performer's creative strategies to modulate or deconstruct available resources, with the validity of the elements residing in the inventive processes rather than the fixed forms themselves.

7 Sheets-Johnstone develops this claim extensively in *The Primacy of Movement* (2011, ch. 1), where she argues that movement is ontologically and epistemologically primary to human experience. She situates this claim within an evolutionary framework: self-movement is the earliest form of animation and awareness across living beings, giving rise to the first sensorimotor meanings (2011, ch. 1, pp. 3–25). This evolutionary continuity underscores her view that human cognition remains rooted in kinesthetic dynamics.

8 Luria argues that movement learning progresses from kinesthetic afferentation of discrete impulses to integrated kinaesthetic structures, such that a single impulse can trigger an entire automated dynamic stereotype (1973, p. 176). Contemporary neuropsychological research supports and extends this view, showing that fluent action sequencing relies on distributed cortical–subcortical networks and anticipatory reorganization (Dahms et al., 2020; Presbrey et al., 2025; Yewbrey and Kornysheva, 2024).

9 Following van Manen (2014, pp. 56–66), *autophenomenology* denotes a self-reflective form of phenomenological inquiry that explores the prereflective, meaning-making dimensions of being-in-the-world through the researcher's own lived experience.

10 According to Ellis et al. (2011), autoethnography is an autobiographical genre of writing and research that displays multiple layers of consciousness “connecting the personal to the cultural” (pp. 739–740).

11 For an in-depth exploration of how somatic awareness and internal sensory feedback function as rhythmic and organizational principles in dance, see *Sensing, Feeling and Action: The Experiential Anatomy of Body-Mind Centering* (Bainbridge Cohen, 2012).

12 An example of conceptual imagination in contemporary dance can be found in *Improvisation Technologies: A Tool for the Analytical Dance Eye*, by Forsythe (2023).

13 See Video 1 for the musical pattern played by the lead drummer adjusting to the movement phrase proposed by the dancer: https://zenodo.org/records/17523374.

14 See Video 2 for the lead drummer cueing the beginning of the music, alerting drummers and dancers that the perofrmance is about to start: https://zenodo.org/records/17522671.

15 See Video 1 for the lead drummer cueing and sustaining the *golobali* until the end of the performance (0:45–1:11): https://zenodo.org/records/17523374.

16 See Video 2 for the lead drummer cueing calls for dance transitions: https://zenodo.org/records/17522671.

17 See an example of the transformation of tempo during a local village celebration recorded south of Bamako by Rainer (2007): https://www.youtube.com/watch?v=D25qj7SS3u8.

18 See Polak and London (2022) for an extended discussion of the relationship between tempo and form in Malian drumming genres.

19 Luria describes four conditions for movement performance: first, kinesthetic afferentation, sensory information from the body to the brain; second, a system of spatial coordinates, vestibular awareness and cutaneous-kinesthetic sensation; third, the release of chains of consecutive movements; and fourth, motor task execution (Luria, 1973, pp. 35–37).

20 An example of how dance improvisation foregrounds sensation as a point of departure can be found in Steve Paxton's *Material for the Spine*, section titled “A Technique for Improvisation” (Paxton et al., 2008). This sensory-based approach is also evident in Paxton's meditation exercise *Small Dance* (1972), which explores stillness and internal movement awareness (The AMAM, 2020).

21 The *prance* emulates a horselike gait, as the dancer's feet “peel up from the dance floor while pushing through every part of the foot” (BBC Arts, 2015). See video of the prance movement here: https://www.bbc.co.uk/programmes/p02pdf7x#:~:text=Martha%20Graham%20used%20a%20lot,Monin%20from%20Phoenix%20Dance%20Theatre.
